# The Three-Dimensional Ultrastructure of the Human Alveolar Epithelium Revealed by Focused Ion Beam Electron Microscopy

**DOI:** 10.3390/ijms21031089

**Published:** 2020-02-06

**Authors:** Jan Philipp Schneider, Christoph Wrede, Christian Mühlfeld

**Affiliations:** 1 Institute of Functional and Applied Anatomy, Hannover Medical School, Carl-Neuberg-Straße 1, 30625 Hannover, Germany; wrede.christoph@mh-hannover.de (C.W.); muehlfeld.christian@mh-hannover.de (C.M.); 2 Biomedical Research in Endstage and Obstructive Lung Disease Hannover (BREATH), Member of the German Center for Lung Research (DZL), Hannover Medical School, Carl-Neuberg-Straße 1, 30625 Hannover, Germany; 3 Research Core Unit Electron Microscopy, Hannover Medical School, Carl-Neuberg-Straße 1, 30625 Hannover, Germany

**Keywords:** lung, alveolus, type 1 alveolar epithelial cell, type 2 alveolar epithelial cell, focused ion beam scanning electron microscopy, 3D reconstruction

## Abstract

Thin type 1 alveolar epithelial (AE1) and surfactant producing type 2 alveolar epithelial (AE2) cells line the alveoli in the lung and are essential for normal lung function. Function is intimately interrelated to structure, so that detailed knowledge of the epithelial ultrastructure can significantly enhance our understanding of its function. The basolateral surface of the cells or the epithelial contact sites are of special interest, because they play an important role in intercellular communication or stabilizing the epithelium. The latter is in particular important for the lung with its variable volume. The aim of the present study was to investigate the three-dimensional (3D) ultrastructure of the *human* alveolar epithelium focusing on contact sites and the basolateral cell membrane of AE2 cells using focused ion beam electron microscopy and subsequent 3D reconstructions. The study provides detailed surface reconstructions of two AE1 cell domains and two AE2 cells, showing AE1/AE1, AE1/AE2 and AE2/AE2 contact sites, basolateral microvilli pits at AE2 cells and small AE1 processes beneath AE2 cells. Furthermore, we show reconstructions of a surfactant secretion pore, enlargements of the apical AE1 cell surface and long folds bordering grooves on the basal AE1 cell surface. The functional implications of our findings are discussed. These findings may lay the structural basis for further molecular investigations.

## 1. Introduction

Type 1 alveolar epithelial (AE1) and type 2 alveolar epithelial (AE2) cells form the epithelial lining of alveoli in the *human* lung. Both are essential for normal lung function: AE1 cells cover the majority of the alveolar surface with thin cytoplasmic extensions that participate in a very thin blood gas barrier and AE2 cells serve as the regeneration source for the alveolar epithelium and secrete the pulmonary surfactant, which lowers the surface tension at the air liquid interface and, thus, prevents alveolar collapse [1], see also [2]. In the past, it was suggested that these cells may show complex morphological properties, such as having more than one apical surface for serving different alveoli or branching of AE1 cells [3,4]. Sirianni et al. [5] described basolateral microvilli on AE2 cells for contacting interstitial fibroblasts and AE1 cells that extend both above and underneath AE2 cells. In a recent study, three entire AE1 cells were modeled in three dimensions (3D) by manual segmentation of a serial block-face (SBF) scanning electron microscopic data set of a *human* lung sample. Both the branching capability and the chance of having more than one apical surface could be confirmed for AE1 cells by this method. Additionally, the 3D data set revealed that different branches of the same cell can form cellular junctions between each other [6]. During this study the questions arose how the sites of contacts both between AE1 and AE1 and between AE1 and AE2 cells as well as the lateral borders of AE2 cells are configured in 3D. The SBF data suggested a complex morphology of AE1/AE2 cell contacts, variable overlap of adjacent AE1 cells and the existence of numerous microvilli that may appear clustered in small niches of the basolateral AE2 cell surface, findings in line with those of Sirianni et al. [5] and Mercurio and Rhodin [7,8]. A special 3D organization of the contact sites may be of relevance for the mechanical stability (or behavior) and integrity of the alveolar epithelium during breathing or the migration of cells from the interstitial space into the alveolus. Knowledge about the detailed 3D organization of the basolateral cell membrane of AE2 cells may help to understand the crosstalk between AE2 and other cells of the septal wall.

While the SBF scanning electron microscope (SEM) used by Schneider et al. [6] has the advantage that it can image rather large fields of view (FOV) and, thus, volumes with entire AE1 cells, it has to live with a compromise in lateral and axial resolution. A high resolution combined with a large FOV tremendously increases the scanning duration (see [9]) and/or may lead to beam damage at the specimen because of exposure to the electron beam. The latter is in particular significant for lung samples because of the small amount of conducting tissue in the epoxy resin block since the lung primarily “consists of air”. Using lower section thicknesses fortifies this problem, cf. [10], so the minimal section thickness as the major determinant of the z-resolution is also a limiting factor of SBF SEM. Thus, for 3D evaluation of structural details, such as cellular junctions or microvilli, focused ion beam (FIB) SEM with both a higher x-/y- and in particular higher z-resolution, appeared to be more appropriate (consider the dimensions of microvilli and the section thicknesses in the aforementioned studies: 80 nm in Schneider et al. [6], 100 nm in Sirianni et al. [5] and unknown in Mercurio and Rhodin [7,8], for review of SBF SEM and FIB SEM, see [10,11,12]). As a consequence, after having reconstructed entire AE1 cells by SBF SEM, we conducted the current study to explore the complexity of the AE1 cell contact sites and the structure of the basolateral AE2 cell membrane using FIB SEM. It was expected that the new insights into AE1 and AE2 cell ultrastructure would enhance our functional understanding of the alveolar epithelium.

## 2. Results

### 2.1. Generation of the Data Set for 3D Reconstructions

After export, the data set comprised 2297 images with a size of 6663 × 4635 px^2^ and a pixel size of 5 nm. Based on the number of images and the penetration depth into the z-direction, an average section thickness of 9.94 nm could be calculated, which is less than 1 % deviation from the desired thickness of 10 nm. Since the milling process and, thus, the section thickness, is an undulation around an average and because the system needs a stabilization period after initiation of the image acquisition, the first 139 images were not accounted for in the calculation. This cut off was determined by qualitative inspection of the z-advance at the beginning of the data set.

### 2.2. Segmentation and 3D Reconstructions

**Figure 1:**
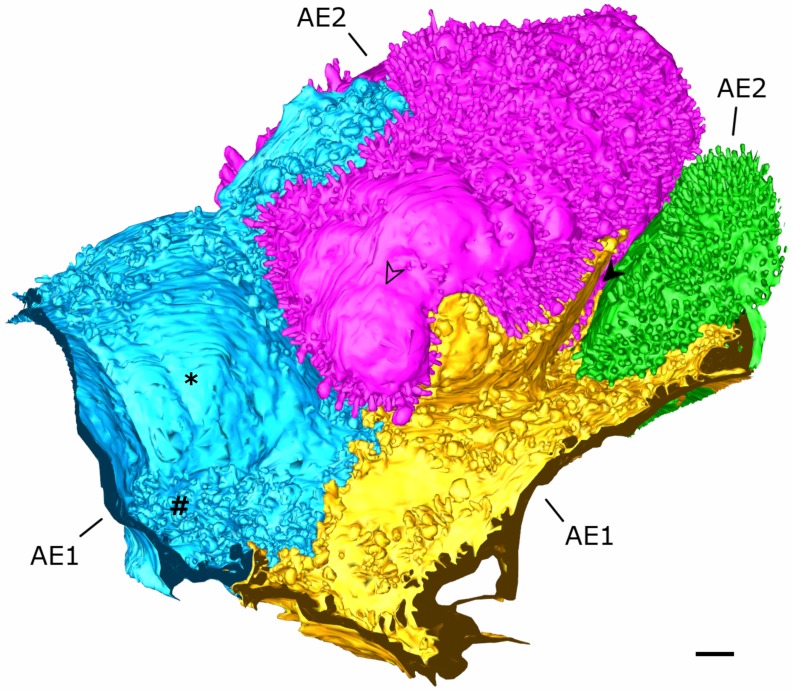
**Overview of the 3D model.** The figure shows an overview of the 3D model from an alveolar viewing direction. The model includes an almost complete type 2 alveolar epithelial (AE2) cell (pink) and parts of another AE2 cell (green) as well as two type 1 alveolar epithelial (AE1) cell domains (blue and yellow), which are labeled as AE2 and AE1, respectively. The filled arrowhead indicates a deep recess between the pink and green AE2 cells as well as the yellow AE1 cell. The AE2 cells show abundant microvilli on their surfaces but also plain parts (empty arrowhead on the pink AE2 cell, for example). The luminal AE1 cell surface may also be plain at a certain spot (asterisk) or enlarged somewhere else (hash key). Scale bar: 1 μm.

Two AE1 cell domains and parts of two AE2 cells in immediate topographic relationship were segmented and reconstructed in 3D for the current work. The part of the dataset underlying the segmentations comprises the images 378 to 1624 of the dataset. A global overview of the models is given in Figure 1 and Table 1 indicates how many outlines per AE1 cell domain and AE2 cell were manually segmented (12,623 in total). It should be noted at this point, that AE1 cells are obviously capable of making cellular junctions with themselves [6], so it cannot be excluded that the two “AE1 models” are parts of one and the same cell. To account for this, we stick to the term AE1 cell domain(s) instead of AE1 cell(s), when we refer to the particular models. Apart from a few, very small portions, the pink AE2 cell is completely included in the dataset and, thus, the model also comprises almost the entire AE2 cell. The small “defects” because of missing portions can be seen in Figure 3.

### 2.3. Epithelial Surface

**Figure 2:**
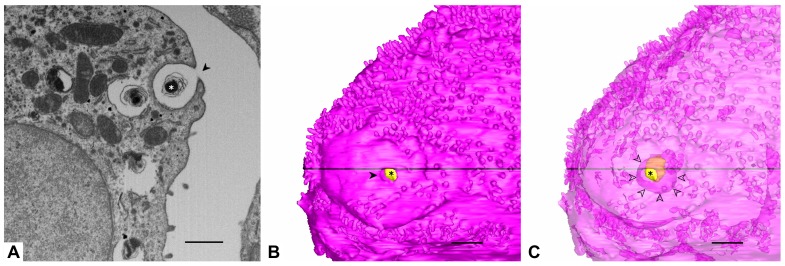
**Luminal AE2 cell surface and surfactant secretion pore.** (**A**) Detail from image 884 which shows an open surfactant secretion pore (arrowhead) and remnants of a lamellar body inside (asterisk). In this image the opening is much smaller than the profile diameter of the vesicle under secretion. (**B**) 3D model of the AE2 cell with the secretion pore (arrowhead) and the remnants of the lamellar body inside (yellow, asterisk). Note the plain cell surface in this area. (**C**) Same image content as in B but with a transparent cell body, which reveals the entire remnant of the lamellar body and indicates the size of the secretory vesicle (circumference marked by arrowheads), the projection of which is much larger than the secretion pore. Scale bars: 1 μm. The black lines in B and C indicate the position of the section plane of A.

**Figure 3:**
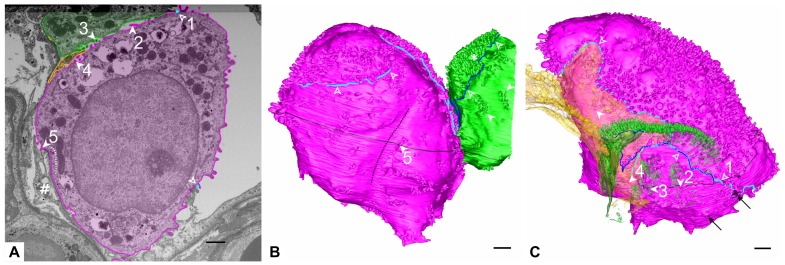
**Basolateral surface of AE2 cells.** (**A**) Segmented electron micrograph (image 988 of the data set). The micrograph shows the segmentations of the pink and green AE2 cell as well as of the yellow AE1 cell domain with opaque outlines and transparent filling. Microvilli can be found at different sites of the AE2 cell, three of them are indicated on the micrograph: towards the adjacent green AE2 cell, which also shows microvilli at this site (filled arrowheads 2 and 3), towards the yellow AE1 cell domain (filled arrowhead 4), towards an interstitial cell (hash key) through a hole in the basal lamina (filled arrowhead 5). Turquoise circles indicate the lateral luminal cell border of the pink AE2 cell (empty arrowheads). The position at empty arrowhead 1 is found in the 3D model in C. The dotted line indicates the profile of the groove depicted in B. (**B**) 3D Model of the pink and green AE2 cells. The position of the segmented electron microscopic (EM) plane in A is indicated in black and the lateral luminal cell borders of the pink and green AE2 cells are indicated by turquoise and blue delineations, respectively (empty arrowheads; note that the positions do not correspond to the empty arrowheads in A). The pink AE2 cell has only one luminal surface. Note the abrupt appearance of a dense microvilli lawn on the luminal surface in some areas (asterisk). Filled arrowhead 5 indicates the region of the corresponding arrowhead 5 in A. Note also the surrounding basolateral microvilli, which may appear in larger groups. Two sites of basolateral groups of microvilli on the green cell are indicated (unnumbered filled arrowheads). Note the long and mostly smooth groove of the pink AE2 cell surface (dotted line). (**C**) Opaque 3D model of the pink AE2 cell and transparent models of the green AE2 cell and the yellow AE1 cell domain. The position of the segmented EM plane in A is indicated in black. The transparency of the yellow AE1 cell domain and the green AE2 cell enable visualization of intercellular microvilli. Numbered arrowheads correspond to the arrowheads in A: Filled arrowhead 2 and 3: Microvilli between the pink and green AE2 cells. Filled arrowhead 4: AE2 microvilli towards the yellow AE1 cell domain. The unnumbered filled arrowhead indicates another region with AE2 microvilli under the yellow AE1 cell domain. The lateral luminal AE2 cell borders are indicated by turquoise and blue delineations (cf. B). Empty arrowhead 1 refers to the corresponding empty arrowhead in A. Black arrows indicate the small parts of the cell where the cell extends beyond the available dataset. Scale bars: 1 μm.

The apical AE2 cell surface exhibits smooth areas surrounded by microvilli as described previously [1]. The pink cell has two larger plain areas and one of them shows an open surfactant secretion pore (Figure 2). (At this stage of exocytosis) the pore has a smaller diameter than the vesicle under secretion, cf. [1]. The green AE2 cell shows a larger plain surface area in the recess between the adjacent AE2 cells (Figure 1). Also the basolateral AE2 cell surface shows abundant microvilli. In contrast to the luminal microvilli, they may appear clustered in small groups, located in small niches of the cell surface. These microvilli are found both between adjacent epithelial cells (AE2 and AE2 or AE2 and AE1), but also at gaps in the basal lamina, where they may reach interstitial cells (Figure 3). They are even found above a continuous basal lamina (not shown).

**Figure 4:**
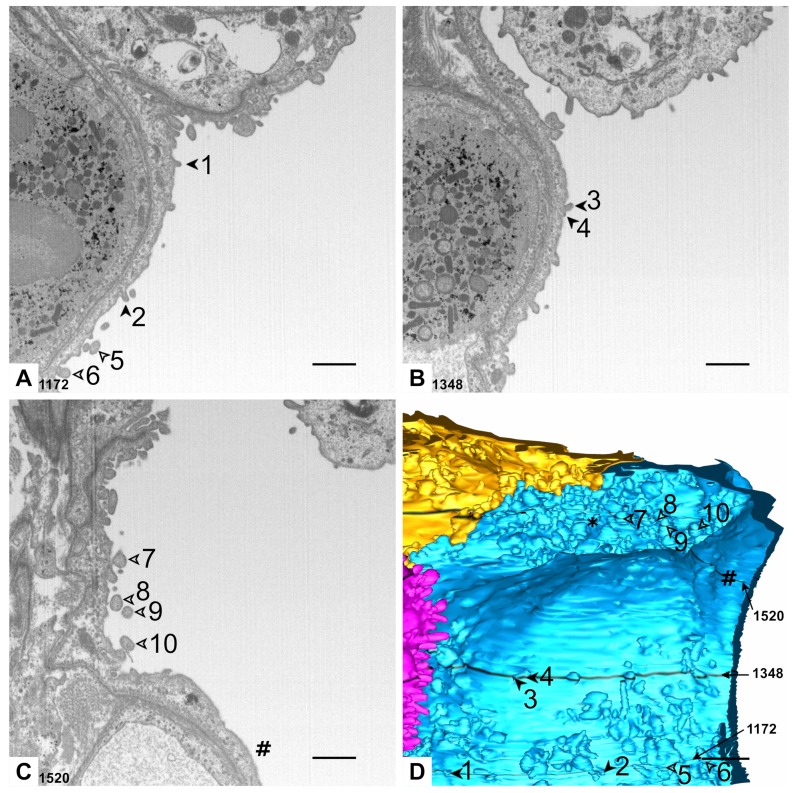
**Luminal surface of AE1 cells.** (**A–C**) Images 1172, 1348 and 1520 of the dataset. Filled arrowheads 1 and 2 (**A**) indicate profiles of microvilli. Filled arrowheads 3 and 4 (**B**) indicate microvilli with a shared basis. Empty arrowheads 5 and 6 (**A**) and 7–10 (**C**) indicate profiles of plasma membrane protrusions filled with cytoplasm. The hash key indicates a region with smooth AE1 luminal surface. (**D**) Detail of the 3D model. Arrowheads and hash key refer to the particular profiles in A–C. The particular section planes 1172, 1348 and 1520 are labeled and emphasized by black color in the model. The different thickness of these black markings is caused by different segmentation intervals in these regions. Note the smooth surface of the cell in the center of the image and the surface enlargements around. Some of them seem to be concentrated in a surface cavity (asterisk). Scale bars: 1 μm.

**Figure 5:**
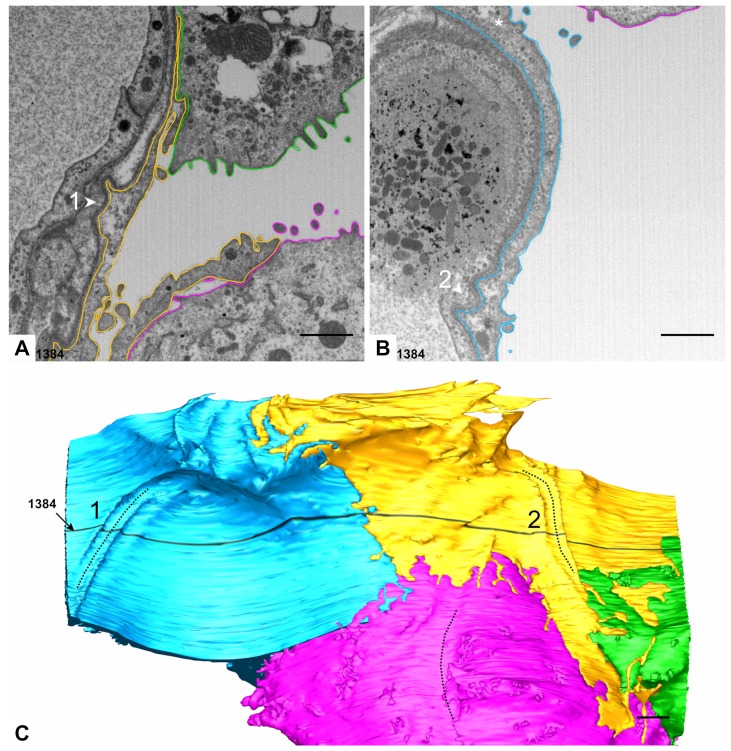
**Basolateral surface of AE1 cells.** (**A**) Detail of the segmented EM plane 1384 including parts of the yellow AE1 cell domain. At the basal side small folds may appear which create a groove-like profile with the concavity pointing towards the interstitial space. The groove beyond the epithelium is filled with extracellular matrix (arrowhead 1). (**B**) A similar situation is found at the basal side of the blue AE1 cell domain, but here the groove is filled with capillary endothelium (arrowhead 2). Note the densely packed caveolae, which appear to be predominantly located in the basal compartment of the cell (asterisk). (**C**) View on the basal side of the 3D model. The model reveals that indeed the AE1 cell domains form grooves on their basal side which are bordered by cellular folds. The groove in the AE2 cell surface, which has been already described in Figure 3, is also indicated by a dotted line. The EM plane 1384 is indicated by black color. Scale bars: 1 μm.

The AE1 cell domains also exhibit a combination of smooth surface areas with surface enlargements (Figure 1 and Figure 4). In contrast to the AE2 cells, this enlargement is realized primarily by protrusions of the plasma membrane filled with cytoplasm. However, some microvilli are found here as well, which can be identified by their regular structure and inner architecture of the cytoskeleton. Some microvilli may share a common basis and some seem to rest on surface protrusions. Both AE1 cell domains show grooves bordered by cellular folds on their basal surface. These grooves stretch across the cells in a relatively orientated fashion along space. Beneath the groove of the yellow AE1 cell domain primarily extracellular matrix is found while it is primarily capillary endothelium beneath the blue cell domain (Figure 5). Additionally, the AE1 cells show abundant caveolae and it is the basal compartment that seems to house most of them (Figure 5B).

### 2.4. Sites of Cell Contacts

**Figure 6:**
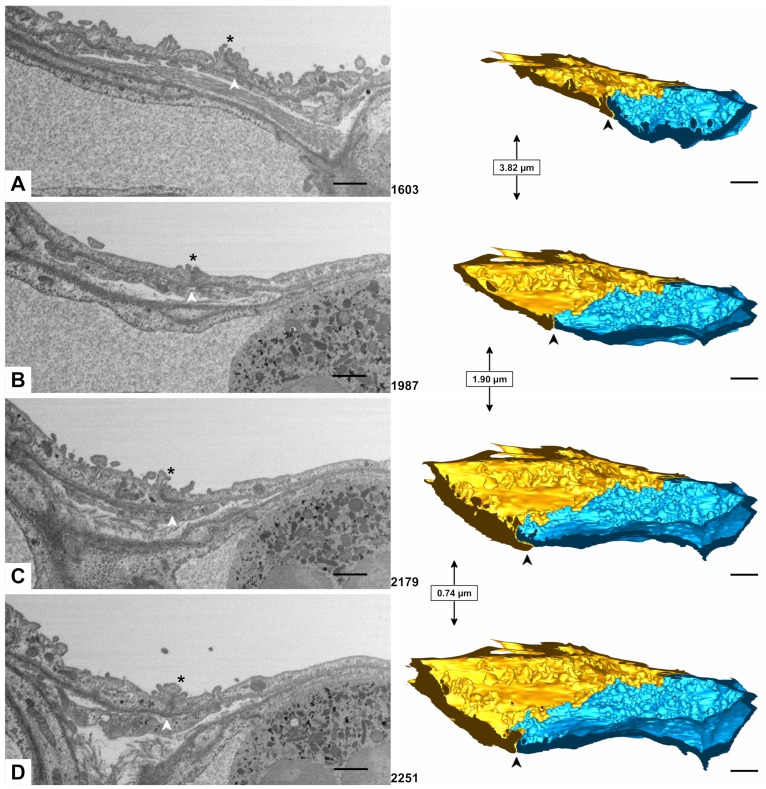
**AE1/AE1 contact.** (**A**–**D**) show the x-plane reconstructions 1603, 1987, 2179 and 2251 of the data set (lateral view on the dataset) on the left side of the panel and a clipped model of the yellow and blue AE1 cell domains on the right side of the panel. The transect plane in the foreground corresponds to the EM plane on the left side. The site of cell contact is indicated by arrowheads. The distance between the different transect planes is indicated in micrometers between the models. Note how the yellow AE1 cell domain slips under the blue cell domain in A and C while the cell domains in B and D meet each other just edge to edge. Note also the bulging of the cell domains into the alveolar lumen at the contact site (asterisks on the EM images). Scale bars: 1 μm.

**Figure 7:**
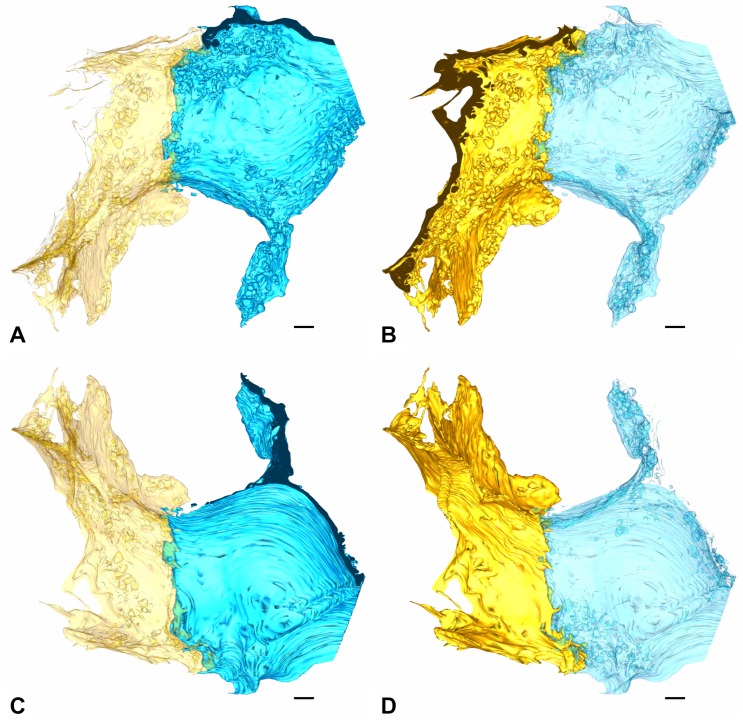
**AE1/AE1 contact.** (**A**,**B**) show the yellow and blue AE1 cell domain with their alveolar surfaces. On each image one of both cell domains is shown transparently (A: yellow, B: blue) to visualize overlap. Images (**C**,**D**) follow the same principle but show the basolateral surfaces. Scale bars: 1 μm.

**Figure 8:**
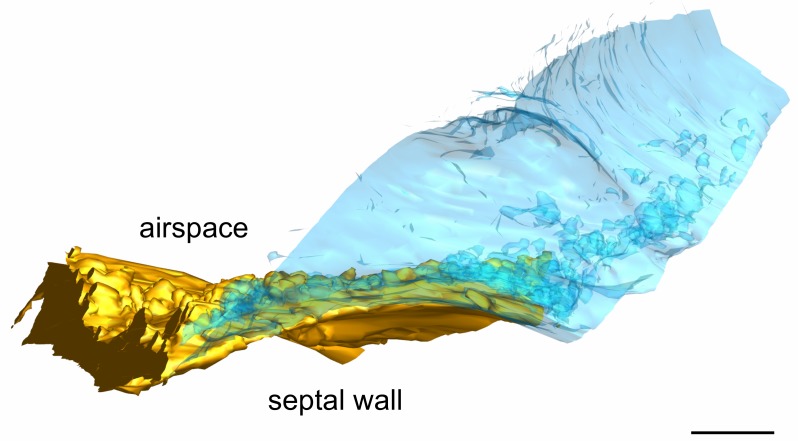
**AE1/AE1 contact.** The image shows the contact of parts of the yellow and blue AE1 cell domains. The blue cell domain in the foreground is displayed transparently to enable visualization of the yellow AE1 cell domain. Note how the yellow AE1 cell domain slips beneath the blue cell domain. Scale bar: 1 μm.

**Figure 9:**
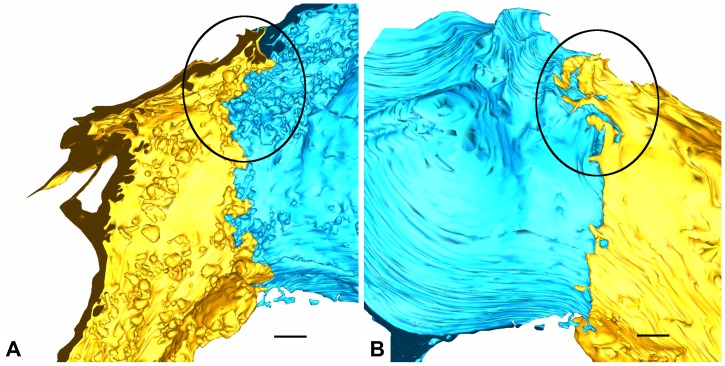
**AE1/AE1 contact.** (**A**,**B**) again show the yellow and blue AE1 cell domain from a luminal (**A**) and a basolateral (**B**) perspective but without transparency. Note the irregular contact site on the abluminal side compared to the luminal side caused by interdigitating cell processes. Compare in particular the region emphasized by the ellipse. Scale bars: 1 μm.

The AE1 cell domains contact each other with or without overlap. At a certain point they may simply prod against each other (edge to edge), while a few micrometers further one cell slips under the other. Directly at the site of contact they may bulge out into the alveolus (Figure 6, Figure 7 and Figure 8). On the basolateral side, cellular processes may interdigitate with the neighbor cell domain, while the luminal edges appear more regular (Figure 9).

**Figure 10:**
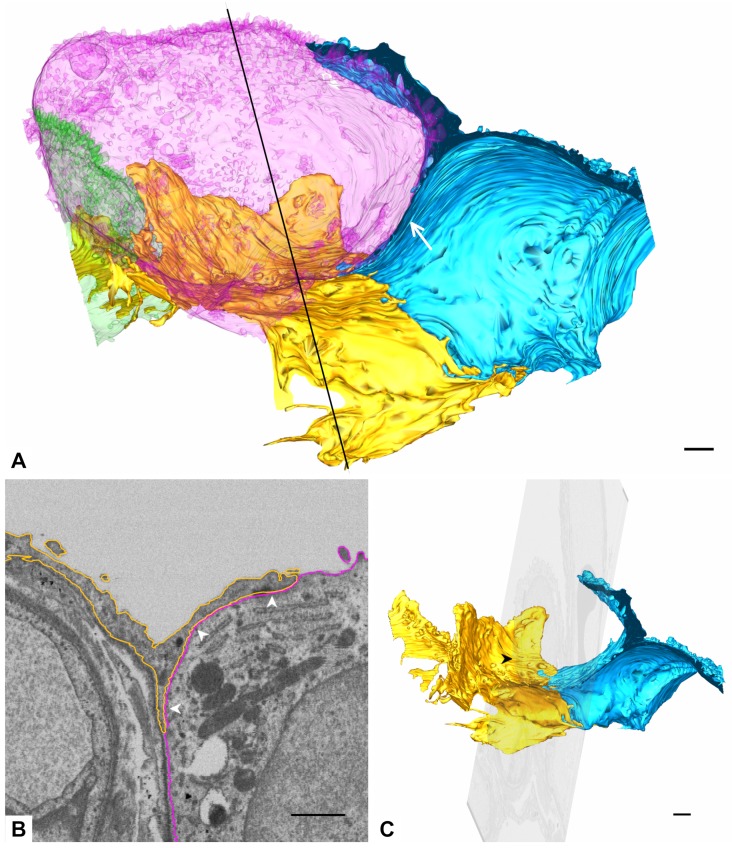
**AE1/AE2 contact.** (**A**) 3D model of the two AE1 cell domains and AE2 cells. AE2 cell models are transparent to enable visualization of the yellow AE1 cell domain behind the AE2 cells. AE1 cells may branch at the edge of an AE2 cell and extend their branches beneath and over the AE2 cell to form a bowl-like structure that enwraps the AE2 cell partially. The yellow AE1 cell domain forms a bowl at the edge of the pink AE2 cell (behind the transparent AE2 cell model). Note also the y-shaped branching of the blue AE1 cell domain and its extension beneath the pink AE2 cell (arrow). The black line indicates the position of the transect image in B. (**B**) Reconstruction of the y-plane (lateral view on the dataset) at the position indicated by the black line in A. The profiles of the yellow AE1 cell domain and the pink AE2 cell are indicated by yellow and pink outlines. Note the y-shaped profile of the yellow AE1 cell domain. The surface of the yellow AE1 cell domain (“inner surface” of the bowl) looked at in A and C is indicated by arrowheads. (**C**) 3D model of the yellow and blue AE1 cell domain transected by the y-plane shown in B. The different angle of view compared to A facilitates the imagination of a bowl formed by the yellow AE1 cell domain. The arrowhead indicates where the image plane transects the bowl of the yellow AE1 cell domain. Scale bars: 1 μm.

**Figure 11:**
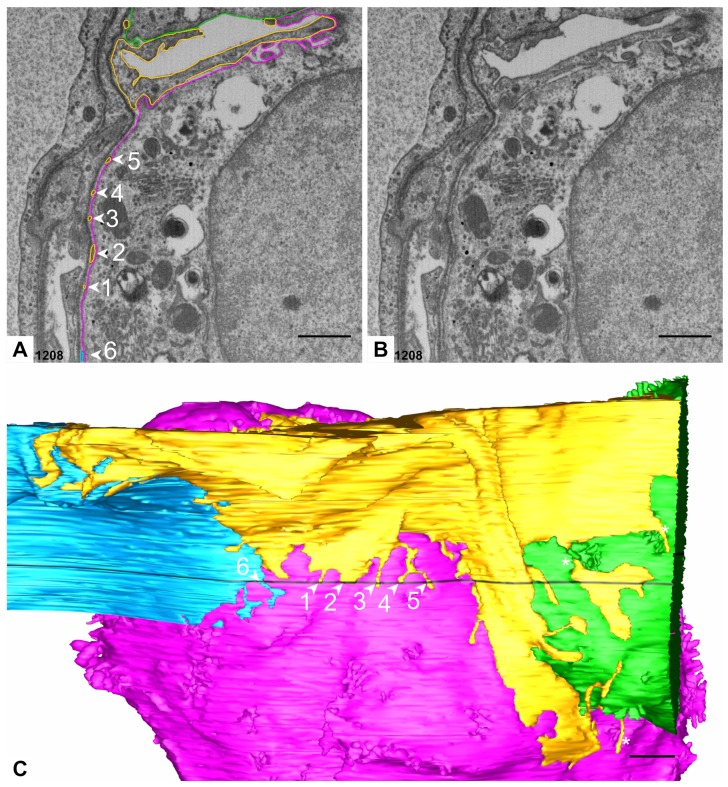
**AE1/AE2 contact.** (**A**) Detail of segmented image 1208. The image shows the segmentation of the pink and green AE2 cells as well as of the yellow and blue AE1 cell domain. Note the very small profiles of the yellow AE1 cell domain (arrowheads 1–5) and the small profile of the blue AE1 cell domain (arrowhead 6). These will probably be overseen if only single images are investigated. If they are recognized, it will almost be impossible to assign them to different cells (compare B). Only the sequence of images reveals their belonging. (**B**) The same EM image as in A but without segmentations. (**C**) 3D Modell of the yellow and blue AE1 cell domain as well as the pink and green AE2 cell with a view on the basolateral cell surfaces. The 3D model reveals that the labeled profiles in A belong to small and thin AE1 processes that crawl along the surface of the pink AE2 cell. The numbered arrowheads correspond to the arrowheads in A. Note also the processes of the yellow cell domain along the green AE2 cell. Some of the processes are found in close proximity to AE2 cell microvilli (asterisks). Scale bars: 1 μm.

At the contact site to AE2 cells, AE1 cells may simultaneously both crawl up and beneath AE2 cells. This is achieved by a y-shaped branching pattern with one branch crawling up and the other branch crawling beneath the AE2 cell. In 3D, this behavior reminds of a bowl partially enwrapping the AE2 cell (Figure 10). Additionally, the AE1 cells may send out thin, finger-like processes into the space between the AE2 cells and the underlying basal lamina. Such processes can be found in close proximity to AE2 microvilli (Figure 11).

**Figure 12:**
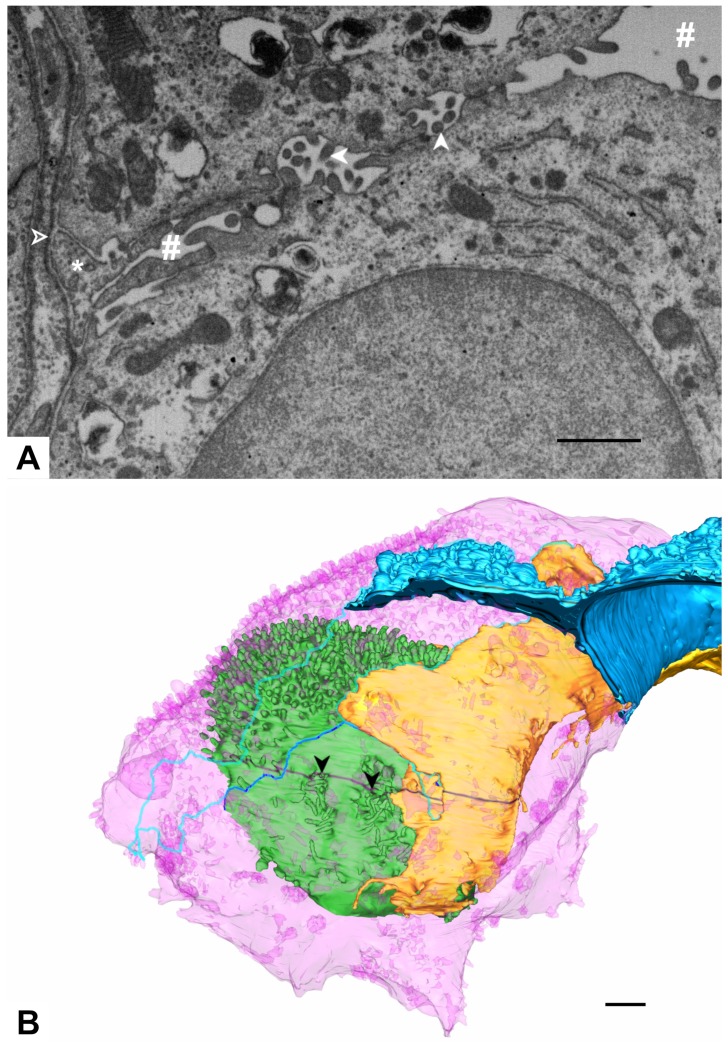
**AE2/AE2 contact.** (**A**) EM image 1112. The image shows the green (top) and pink (bottom) AE2 cell contacting each other. Both cells show niches with microvilli on their basolateral surface facing each other (filled arrowheads). Between both cells also parts of the yellow AE1 cell domain are found (asterisk). The alveolar lumen is indicated by the hash key and the basal lamina by an empty arrowhead. (**B**) 3D model of the pink and green AE2 cell as well as the yellow and blue AE1 cell domains. The image plane of A is indicated in black. Arrowheads correspond to the arrowheads in A. The borders of the luminal/abluminal surface of the AE2 cells are indicated by turquoise (pink cell) and blue lines (green cell) (cf. Figure 3). The pink AE2 cell and its luminal/abluminal border are displayed transparently to visualize the green AE2 cell and yellow AE1 cell domain behind as well as the luminal/abluminal border of the green AE2 cell. Note the well defined niches with microvilli on the basolateral surface of the green AE2 cell (black arrowheads). Scale bars: 1 μm.

The two AE2 cells contact each other edge to edge, with niches of microvilli facing each other (Figure 12).

## 3. Discussion

Volume electron microscopic (EM) techniques for 3D modeling of biological structures, including “conventional” techniques like single sectioning transmission electron microscopy (ssTEM) and “new” techniques like SBF SEM and FIB SEM, have been reviewed extensively recently, e.g., [9,10,11,12,13] and some of them have already been used for 3D reconstructions of lung structure: ssTEM [5,7,8,14]; electron tomography [15]; array tomography [16] and SBF SEM [6,11]. Even FIB SEM has been applied [17,18,19], but the study of Købler et al. was rather focused on testing an alternative method to diamond knife ultramicrotomy and transmission electron microscopic imaging of nanotubes; the review of Ochs et al. gave an outlook on the potential of this technique in lung research; and Hegermann et al. demonstrated a method for correlative light and electron microscopy. Kremer et al. [11] showed a FIB SEM-based reconstruction of a small part of a *human* A549 cell. Here, to our knowledge, we provide the first extensive study of the alveolar epithelial 3D ultrastructure based on FIB SEM in a *human* lung sample. We were able to reconstruct an almost complete AE2 cell with two adjacent AE1 cell domains as well as a part of a neighboring AE2 cell. The model shows detailed reconstructions of the epithelial surface, including a surfactant secretion pore on an AE2 cell, enlargements of the apical AE1 cell surface, long folds bordering grooves on the basal AE1 cell surface, AE1/AE1, AE1/AE2 and AE2/AE2 contact sites, basolateral microvilli pits at AE2 cells and small AE1 processes beneath AE2 cells. The functional relevance of these findings will be discussed below.

AE1 cells seem to be responsible for the majority of fluid transport across the alveolar epithelium [20,21,22]. According to Dobbs et al. [20] different sodium channels (highly selective Na^+^ channel (HSC), non-selective Na^+^ channel (NSC) and cyclic nucleotide-gated channel (CNG)), the cystic fibrosis transmembrane regulator (chloride channel), potassium and water (Aquaporin 5) channels and Na^+^/K^+^ATPases are involved in that. The surface enlargements of the AE1 cell domains shown here may serve as a membrane compartment housing the particular transport proteins and, thus, be the ultrastructural correlate of liquid absorbtion.

Interestingly, when looking at Figure 4, one gets the idea, that the surface in a septal concavity is rich in these surface enlargements, while they seem to be reduced or even absent on convexities. Concave niches are filled with the (liquid) hypophase of surfactant [1,7] and these “puddles” may be the sites where most of the fluid absorption takes place. Microvilli on AE1 cells have been described previously [20] and were also found during *cat* lung development [8,14].

Fixation (cf. the studies of Gil et al. [23], Oldmixon et al. [24] and Oldmixon and Hoppin Jr. [25] or long-term storage can lead to microscopic artefacts. The following arguments, however, indicate that the non-microvillus surface enlargements are a real biological phenomenon: (1) The cytoplasm within these excrescences is quite electron dense, while lytic cell parts regularly appear electron light. (2) The surface enlargements seem to be concentrated at certain spots, while other areas remain smooth. (3) Microvillus-like surface irregularities without obvious microfilaments have also been described by Mercurio and Rhodin [7] in the *cat*. (4) Surface irregularities on AE1 cells can also be seen on some scanning electron micrographs in textbooks or other articles [1,3,5,26,27].

Another explanation for the surface irregularities was given by Mercurio and Rhodin [7], who suggested that they are incorporated into the cell membrane during inflation. This was concluded, because they also found a cell with a rather smooth surface, where they thought that the flatness was caused by trapped air in the airspaces. If this interpretation is true, however, then also the basolateral cell membrane needs membrane reserves, since the apical and basolateral membranes are separated by tight junctions [28]; Sirianni et al. [5] also mentioned the existence of discontinuous tight junctions. Such reserves could be provided by the cytoplasmic folds of the basolateral cell membrane or by the AE1 cell branches beneath the AE2 cells shown by our models.

The basal AE1 cell membrane folds, however, could also serve as mechanical stabilization, i. e., anchoring the epithelium in the septal wall by interdigitation with the interstitium to resist shearing forces. This principle is well established for the skin, where connective tissue papillae or ridges of the dermal papillary layer interdigitate with the epidermis at the dermal-epidermal junction [29] (p. 147).

With respect to AE1/AE1 cell contacts in the *cat* lung, Mercurio and Rhodin [7,8] described the possibility that adjacent cells may show alternating overlap or meet edge to edge. Our data suggest that this is also true for the *human* lung, even if the images and models shown here only demonstrate a clear overlap with the blue cell domain on top of the yellow cell or edge to edge contacts (Figure 6 and Figure 7). What is the functional relevance of this overlap? Overlap per se enhances mechanical stability but additionally enlarges the contact area; in particular it may enlarge the available “contact area” for important cell junctions like the relatively wide (see [1,30]) tight junctions and/or gap junctions, for review, see [31]. The latter have been described to be in close topographic relation to the occluding junctions [30]. Mercurio and Rhodin [7] suggested that the overlap may change during breathing.

With respect to the AE1/AE2 contacts, we could confirm and extend our SBF-based presumption and Sirianni et al’s [5] data that AE1 cells may extend both under and on the AE2 cells. Our data set and model revealed a y-shaped branching pattern in 3D (cf. Figure 2 of [5]) and additionally added that AE1 cells may extend various thin foot processes under AE2 cells.

Branches beneath AE2 cells may serve as membrane reserve during inspiration (see above). This may also be an explanation for the foot processes, but these may also be structural correlate of cellular interaction: Sirianni et al. [5] demonstrated linkage of AE1 and AE2 cells with interstitial fibroblasts via holes in the basal lamina, which seem to be primarily located beneath AE2 cells and Nabhan et al. [32] demonstrated that Wnt signaling between fibroblasts and alveolar epithelial stem cells (a small fraction of distinct AE2 cells) is necessary to maintain their stem cell status. Eventually, the small AE1 processes “look for” those holes for linkage to interstitial fibroblasts. Alternatively, the close proximity of these processes to AE2 microvilli pits in some cases (see Figure 11) could indicate exchange of material (too large for gap junctional transport) from AE1 to AE2 cells or vice versa (or fibroblasts).

These microvilli pits of AE2 cells, however, were also found towards other cell types (i.e. the other AE2 cell or interstitial cells (see Figure 3). Interestingly, they were also found above an intact basal lamina (not shown). Proximity to cells and holes in the basal lamina suggests a role in intercellular communication, material exchange or sensory functions. Localization above a closed basal lamina may be the structural correlate of basal lamina turnover or the initiation of creating a hole for consecutive subepithelial communication.

Summing up, the current FIB SEM study of a *human* lung sample provided new insights into the *human* alveolar epithelial cell morphology and topography. Our model reveals detailed reconstructions of the alveolar epithelial surface, including a surfactant secretion pore on an AE2 cell, enlargements of the apical AE1 cell surface, long folds bordering grooves on the basal AE1 cell surface, AE1/AE1, AE1/AE2 and AE2/AE2 contact sites, basolateral microvilli pits at AE2 cells and small AE1 processes beneath AE2 cells. These data may serve as morphological blueprint for molecular investigations of alveolar epithelial biology.

## 4. Materials and Methods

### 4.1. Sample Preparation

An archival sample of a *human* lung [33], kindly provided by Professor Ewald R. Weibel (Institute of Anatomy, University of Berne, Berne, Switzerland), was used for the current study. The sample was prepared as described previously [6] following a modified protocol from Deerinck et al. [34] to enhance membrane contrast, cf. also [16]:

In brief, the fixed sample was rinsed in 0.15 M HEPES buffer followed by 0.1 M cacodylate buffer and subsequently postfixed by reduced osmium tetroxide (OsO_4_) (1.5 % hexacyanoferrate II, 1 % OsO_4_ in 0.1 M cacodylate buffer) in the dark for half an hour. The sample was then washed in double distilled water (ddH_2_O), infiltrated with 1 % thiocarbohydrazide in ddH_2_O for 20 min and washed again in ddH_2_O before it was once more postfixed by 1 % OsO_4_ in ddH_2_O in the dark for half an hour. After washing in ddH_2_O the sample was block stained overnight in the dark at 4 ${}^{\circ }$C in an aqueous half saturated uranyl acetate solution, followed by a washing step in ddH_2_O and block staining by Walton’s lead aspartate at 60 ${}^{\circ }$C for half an hour. After washing in ddH_2_O, the sample was dehydrated in an ascending series of acetone (70 %, 90 % and 100 %) and finally embedded in Durcupan (Sigma-Aldrich Chemie GmbH, Munich, Germany).

The sample was trimmed and an ultrathin section (60 nm) was generated to look for appropriate regions of interest in a conventional transmission electron microscope (Morgagni 268, FEI, Eindhoven, Netherlands) for imaging with the FIB SEM.

The Durcupan block was mounted in a slotted SEM specimen holder and the sides were covered with conductive silver (Plano, Wetzlar, Germany) before the sample was sputtered with a 20 nm gold layer (Quorum Q150R ES sputter coater; Quorum Technologies Ltd, Laughton, East Sussex, United Kingdom), cf. [17].

### 4.2. FIB SEM Data Set Acquisition

The prepared specimen with the region of interest (ROI) was approached with a Zeiss Crossbeam 540 (Carl Zeiss Microscopy GmbH, Jena, Germany) at 20 kV acceleration voltage. A deposition of platinum and the generation of carbon-highlighted marks on the area of interest enabled adequate handling, tracking, autofocus and autostigmation during the acquisition process. Finally, using the Inlens Secondary Electron (SE) and the Energy selective Backscattered (EsB) detector (grid voltage 800 V), a z-stack of 2297 images, each showing a ROI of 33 μm × 20 μm was generated (pixel size 2 nm, section thickness 10 nm). Image acquisition was performed at 1.5 kV with 1.0 nA using the ATLAS software package (Carl Zeiss Microscopy GmbH, Jena, Germany) accompanying the microscope, cf. [17]. For later segmentations and 3D reconstructions a cropped data set was exported from the ATLAS software package with a pixel size of 5 nm and an Inlens SE to EsB detector ratio of 80 % to 20 %.

### 4.3. Segmentations and 3D Reconstructions

For segmentations and 3D reconstructions 3dmod (part of the iMOD package [35]) was used: After import of the data set, the cells or cell parts of interest were segmented by manually tracing their outlines on the FIB SEM images. As a basic algorithm the structures were segmented on every fourth image. If the complexity of structure and/or the modeling, however, required a narrower segmentation interval, also sections in between the first and fourth image (up to every image) were segmented. The stack of contours was then used by 3dmod to generate 3D models of the segmented structures. For the AE2 cells also the luminal-abluminal border as an approximation of the apical/basolateral surface border was segmented by tracing the luminal edges of the alveolar cell surface through the z-Stack, cf. [6].

### 4.4. Figure Preparation

Figures were prepared in the GNU Image Manipulation Program (gimp 2.10.12; www.gimp.org [accessed on 23 September 2019]) after having created snapshots with 3dmod.

For a general overview of sample preparation techniques and the volume EM workflow, see [10].

## Figures and Tables

**Table 1:** Number of outlines manually segmented for modeling.

Structure	Number of Outlines
AE1 cell domain 1 (yellow)	2439
AE1 cell domain 2 (blue)	2683
AE2 cell 1 (pink)	5772
AE2 cell 2 (green)	1729
Total amount of outlines	12,623

## Abbreviations

The following abbreviations are used in this manuscript:

AE1	Type 1 alveolar epithelial
AE2	Type 2 alveolar epithelial
3D	Three dimensions/-dimensional
SBF	Serial block-face
SEM	Scanning electron microscope/microscopy
FOV	Field of view
FIB	Focused ion beam
EM	Electron microscopic
HSC	Highly selective Na^+^ channel
NSC	Non-selective Na^+^ channel
CNG	Cyclic nucletide-gated channel
ssTEM	Single sectioning transmission electron microscopy
OsO_4_	Osmium tetroxide
ddH_2_	Double distilled water
ROI	Region of interest
SE	Secondary electron
EsB	Energy selective Backscattered
